# Unmasking the Hidden Threat: Invasive Pulmonary Aspergillosis Following Dengue Hemorrhagic Fever in an Immunocompetent Patient

**DOI:** 10.7759/cureus.85084

**Published:** 2025-05-30

**Authors:** Komal Masud, Shaheena Khan, Muzna Mehmood, Nur Ul Ain

**Affiliations:** 1 Medicine, Kulsoom Medical Center, Islamabad, PAK; 2 Biochemistry, Shifa College of Dentistry, Islamabad, PAK; 3 Plastic and Reconstructive Surgery, Rawalpindi Medical University, Rawalpindi, PAK

**Keywords:** antibiotics, aspergillosis, case report, dengue fever, dengue hemorrhagic fever, pulmonary aspergillosis

## Abstract

Dengue hemorrhagic fever (DHF), an arbovirus-borne infection, is endemic in multiple regions across the globe. It has been observed that patients with severe viral respiratory tract infections are highly susceptible to developing a fungal co-infection. We present a similar case of a 27-year-old female diagnosed with DHF, reporting the development of aspergillosis during recovery from dengue fever. The patient presented with persistent high-grade fever, vomiting, hypotension, and body aches. Lab results showed a dropping platelet count and raised inflammatory markers. Dengue serology by enzyme-linked immunosorbent assay (ELISA) was positive. She had on-and-off episodes of shivering for which a malarial parasite smear was ordered, which came back negative. Chest X-ray showed a heterogeneous opacity in the right lower lobe. The patient was started on intravenous (IV) antibiotics (tazobactam + piperacillin and amikacin sulphate) along with IV dexamethasone. Chest physiotherapy and regular nebulization were also done. A bronchoscopy was performed, which was turbid. No acid-fast bacilli were found, but budding yeast cells were identified. The patient was put on voriconazole oral tablet, after which she became afebrile, and her oxygen saturation started stabilizing. There was a significant improvement in lab results and radiological investigations as well. She was discharged after a month of initial presentation, with antifungal medication until the next follow-up. There should be better means of investigation for such apparent serological disturbances, not dependent on invasive tests. Some modalities should be developed that are rapid, specific, and cost-effective.

## Introduction

Pulmonary aspergillosis may be one of the most lethal and unrecognized infections in critically ill patients [1]. On the other hand, dengue hemorrhagic fever (DHF), an arbovirus-borne infection, is endemic in various regions across the globe, including Africa, the Americas, Southeast Asia, the Western Pacific, and the Eastern Mediterranean. It is worth noting that children, the elderly, and patients with comorbidities are more vulnerable and have shown higher mortality rates when affected by dengue fever [2]. Invasive aspergillosis (IA) is an important cause of morbidity and mortality among hospitalized patients in developing countries, though the exact frequency of the disease is not known due to inadequate reporting and facilities to diagnose [3].

Over the last decade, it has become evident that patients with severe viral respiratory tract infections are highly susceptible to developing a fungal co-infection, in particular pulmonary aspergillosis [4]. In DHF, most patients develop fever, malaise, headache, and skin rash in the early febrile stage of dengue virus infection, but few progress to severe disease with bleeding diathesis, plasma leakage, and profound shock [5]. Pulmonary infiltration seen in dengue-infected patients is generally caused by plasma leakage with or without bacterial infection, which, along with pleural effusion, is regarded as the hallmark of pulmonary manifestation in dengue infection [6].

We present a similar case of a 27-year-old female diagnosed with DHF, reporting the development of IA during recovery from dengue fever. Moreover, this report unfolds the classical sequelae of a patient with a severe viral illness, manifesting with respiratory symptoms, progressing to invasive pulmonary aspergillosis (IPA) due to plasma leakage and exacerbated by low white blood cell count and evidenced thrombocytosis.

## Case presentation

A 27-year-old housewife with no significant past medical and surgical history presented to the emergency department of a tertiary care hospital with persistent high-grade fever, vomiting, and body aches. The patient had already been treated by a local general physician (GP) for dengue based on a low platelet count. It was her seventh day of illness, and lab results showed that her platelet count had started dropping again. She also complained of on-and-off episodes of shivering for which the GP had ordered a malarial parasite (MP) smear, which came back negative.

The patient was a resident of a densely packed colony in Rawalpindi, Pakistan, and presented during the month of August, which signified that she came from a geographical zone at high risk for dengue infection [7].

**Figure 1:**
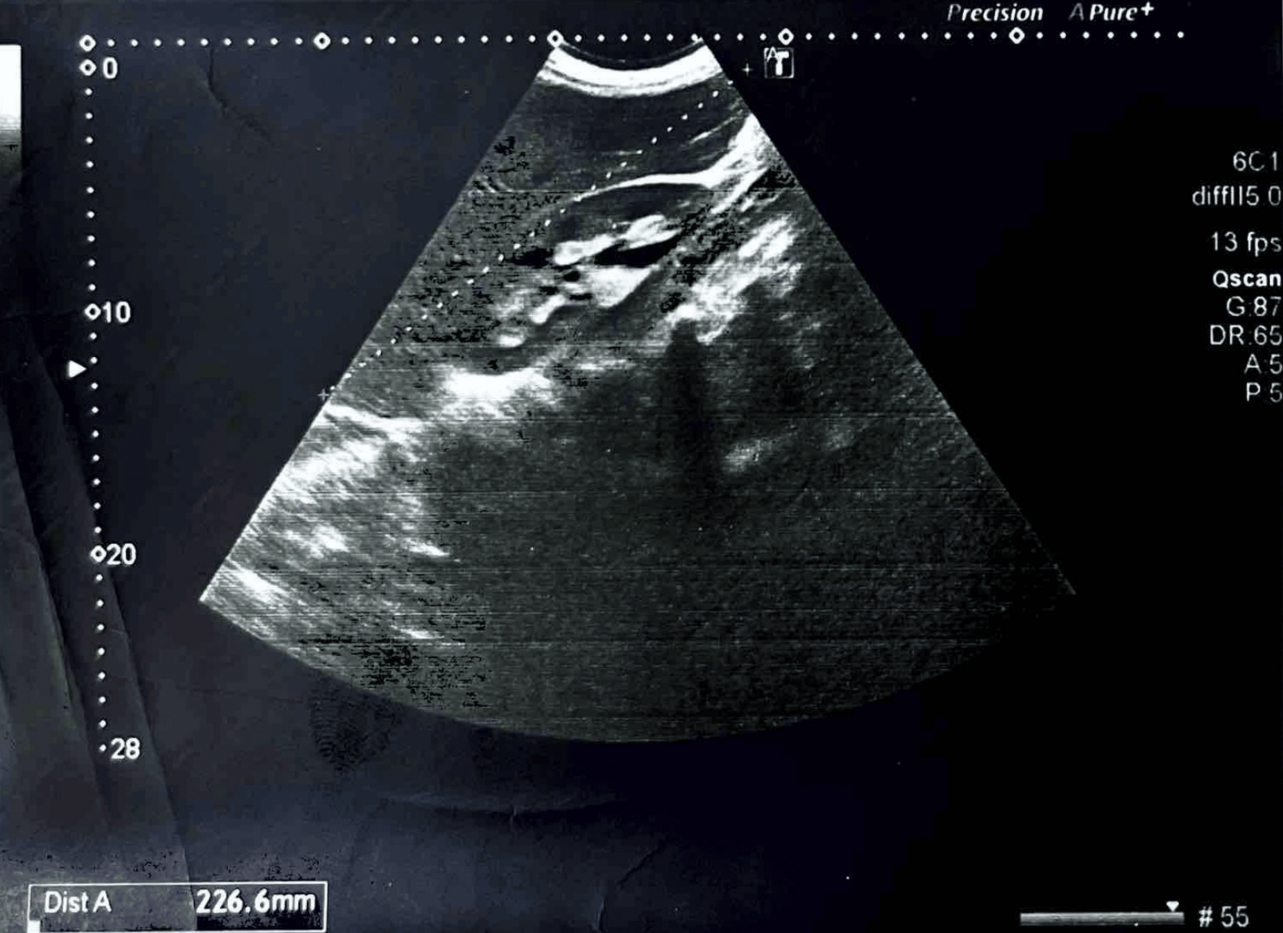
Ultrasound of the abdomen.

She was febrile and hypotensive at the time of presentation and was admitted to the department of infectious diseases (DID) with a suspicion of DHF in the isolation ward. Ultrasound for dengue showed mild free fluid in the pelvic cavity and mild bilateral pleural effusion, with a mild splenomegaly (13 cm) (Figure 1). Posteroanterior (PA) chest X-ray (CXR) showed a heterogeneous opacity noticed in the right lower lobe with cardiomegaly. The patient denied contact with any tuberculosis (TB)-positive patient. Laboratory investigations were also performed. Her C-reactive protein (CRP) was raised to 183.14 mg/L with D-dimer at 1006 ng/ml.

On day two, her hemoglobin dropped from 9.2 g/dl to 7.4 g/dl, and her platelet count decreased from 90,000 to 75,000. Dengue serology nonstructural protein 1 (NS1) antigen via enzyme-linked immunosorbent assay (ELISA) was positive, meeting the WHO criteria for confirmed dengue infection. The diagnosis was further supported by the clinical presentation during the high-incidence dengue season in a known endemic area, alongside laboratory features including thrombocytopenia and raised CRP, consistent with DHF. The patient complained of palpitations, for which an electrocardiogram (ECG) was performed, which showed sinus tachycardia. Two units of red cell concentrates (RCC) were transfused on the second and fourth day of admission, which increased the hemoglobin (Hb) to 8.8 g/dl and platelets to 90,000. Since the critical period of the disease had ended, the CRP also improved. However, there were still several episodes of shivering recorded for which artesunate injection was administered prophylactically due to recurring chills and shivering episodes, as a precaution against possible missed or concurrent malarial infection - a clinical decision common in third-world dengue-endemic regions like Pakistan, where malaria co-infection cannot be immediately ruled out, due to lack and unreliability of resources, even with an initially negative smear. By the fifth day, the patient was vitally stable. Lab investigations also showed progressive improvement, which led to her discharge (Table 1).

**Table 1:** Laboratory parameters on presentation during the first hospitalization. CRP: C-reactive protein; Hb: hemoglobin; Hct: hematocrit; WBC: white blood cells.

		Day 01	Day 02	Day 03	Day 04	Normal values
Blood count	Hb (g/dL)	9.2	7.4	8.3	8.4	12.0-16.0
	Hct (%)	28	24.5	26.4	26.1	36-46
	WBC (x1000/mm^3^)	8.8	7.0	10.3	9.5	4,000-11,000
	Platelet (x1000/mm^3^)	90	75	92	102	140,000-425,000
Inflammatory markers	CRP (mg/L)	183.14	-	-	39	0-5
	D-dimer (ng/ml)	1006	-	-	-	Less than 250

However, two days after the discharge, the patient presented again in the emergency department of another tertiary care hospital with a respiratory rate of 66 bpm, severe intercostal pain, vomiting, apprehension, and worsened episodes of chills. She was not maintaining saturation at room air, so supplementary oxygen was attached at 9 liters. Urgent arterial blood gases were sent for reporting, which were deranged (pH = 7.543, bicarbonate = 15.6 mmol/L). None of the other laboratory investigations fell within normal limits as well.

Infective markers were also ordered, which showed a raised CRP level of 273 mg/L, procalcitonin of 76.70 μg/L, erythrocyte sedimentation rate (ESR) of 140 mm/h, and lactate dehydrogenase (LDH) of 692 U/L. Serum total iron-binding capacity (TIBC) and peripheral film were within normal limits, but with low iron levels of 17 μg/dL. Dengue IgG, IgM, and NS1 were all negative. The patient was admitted to the medical ward with oxygen support. Her echo showed an ejection fraction of 65% with trace tricuspid regurgitation and severe pulmonary hypertension for which bosentan monohydrate was started orally.

The patient was started on antibiotics and initially reacted to vancomycin, so she was shifted to injection tazobactam + piperacillin and amikacin sulphate along with injection dexamethasone. Furthermore, the patient had no urine output for more than 12 hours, for which injection furosemide was inculcated in the treatment chart twice daily. Chest physiotherapy and regular nebulization were also considered.

Blood culture and sensitivity showed no growth of bacterial pathogens. Autoimmune profiling was also performed for the patient, but all the parameters were negative. Her potassium was replaced, and one unit of RCC was transfused, after which her Hb rose to 11 g/dl and platelet count was 514,000, on the eighth day of admission. Results for urine routine examination (R/E) signified the presence of leukocyte esterase, for which meropenem injection was also added to the regimen. Arterial blood gases, along with other routine laboratory investigations, were carried out daily, which showed slight improvement.

At the time of worsening respiratory symptoms, bacterial pneumonia and tuberculosis were high on the list of differentials. A CXR showed atypical features without cavitation or lobar consolidation. Acid-fast bacilli (AFB) smears and Gene Xpert tests were negative for TB. Additionally, blood and sputum cultures showed no bacterial growth, and the absence of viral pneumonitis markers or contact history supported ruling out other viral etiologies. Persistence of high inflammatory markers and a lack of response to broad-spectrum antibiotics prompted further investigation for a fungal etiology.

**Figure 2:**
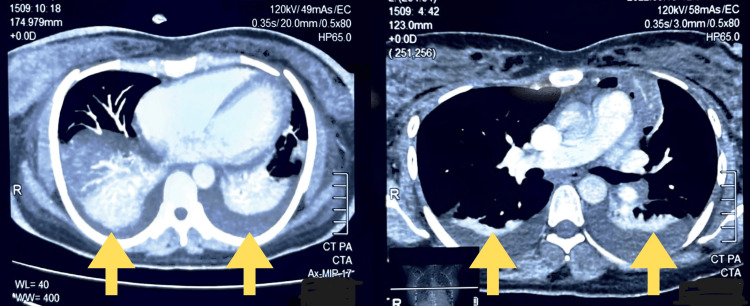
High-resolution computed tomography (HRCT) showing bilateral multifocal areas of dense patches of ground glass opacities with surrounding consolidative changes and moderate pleural effusion and left-sided cavitatory lesions. Keeping in view the patient’s history of dengue hemorrhagic fever, these findings are suggestive of pulmonary hemorrhage with superadded bacterial pneumonia. Yellow arrows point to pleural effusion.

A CT scan of the chest with contrast was performed, which showed evidence of pulmonary embolism, leading to bilateral pulmonary effusion. At the time of pulmonary embolism and bilateral effusion, fungal infection was increasingly suspected but not yet confirmed. The high inflammatory markers, refractory hypoxemia, and sterile cultures had prompted the clinical team to consider fungal causes, particularly invasive pulmonary aspergillosis, which was later supported by bronchoscopy findings. A pleural tap was tried on the patient three times but failed. A lower extremity venous compression exam (bilateral legs) was performed to assess deep venous thrombosis (DVT), which showed no sonological evidence of DVT. Low molecular weight heparin (Clexane) was started along with injections of levofloxacin and linezolid. Echo showed trivial/trace tricuspid regurgitation, a thin rim of pericardial effusion, and normal right ventricular (RV) systolic function. Ultrasound further showed hepatomegaly (liver enlarged to size 163 mm) with no free fluid seen in the abdomino-pelvic cavity or the pleural cavity. Celiac profile and IgA levels of the patient were within normal limits (Figure 2).

On day 16, tracheal lavage for potassium hydroxide (KOH) preparation, AFB stain, and gram stain were performed, which showed no signs of any causative organism. Culture and sensitivity sample of the tracheal lavage indicated no growth of bacterial pathogens. The patient and attendants were again asked about their personal and family history of TB, but they denied it. On microscopy (fluorescent), AFB smear was negative, and a Gene Xpert *Mycobacterium tuberculosis*/rifampicin resistance (MTB/RIF) Ultra test also yielded negative results (Table 2).

**Table 2:** Laboratory parameters on presentation during the second hospitalization. aPTT: activated partial thromboplastin time; CRP: C-reactive protein; Hb: hemoglobin; HCO3: bicarbonate; LDH: lactate dehydrogenase; pH: balance of acid/base; pO2: partial pressure of oxygen; PT: prothrombin time; WBC: white blood cells.

		Day 01	Day 04	Day 11	Day 19	Day 26	Normal values
Blood count	Hb (g/dL)	7.4	8.8	12.2	11.2	9.8	12.0-16.0
	WBC (x1000/mm^3^)	8.62	5.02	23.6	15.2	12.6	4,000-11,000
	Platelet (x1000/mm^3^)	88	100	496	334	629	140,000-425,000
Liver function	Bilirubin (mg/dL)	3.5	1.8	1.5	0.9	0.6	0.3-1.2
	Albumin (g/dL)	2.0	2.3	-	2.8	2.8	3.3-4.5
Renal function	Creatinine (mg/dL)	0.45	0.41	0.3	0.5	0.5	0.55-1.02
Coagulation profile	PT (sec)	17.51	13.66	18.49	14.05	-	10-14
	aPTT (sec)	51.18	30.07	28.41	38.80	-	28-42
Inflammatory marker	CRP (mg/L)	273	61.1	99	-	24.7	0-5
	D-dimer (ng/mL)	626	-	-	-	-	Less than 250
	Procalcitonin	76.70	-	0.75	-	0.12	Less than 0.5
	LDH (U/L)	692	-	-	313	244	50-460
Serum electrolytes	Potassium (mEq/L)	3.1	2.6	3.3	2.6	4.6	3.5-5.5
	Calcium (mg/dL)	6.4	7.5	7.8	8.02	8.59	8.4-10.2
Arterial blood gas	pH	7.529	7.499	7.595	7.602	7.39	7.35-7.45
	pO2 (mmHg)	98	42	45	125	90	85-100
	HCO3 (mmol/L)	19.3	25.1	29.5	32.4	25.3	22-26

On the 18th day of admission, a bronchoscopy was performed. Fluid R/E was found to be turbid with coagulum. No AFB were seen, but budding yeast cells were identified. A cytology report for bronchial aspirate showed fungal hyphae and conidia. However, beta-D-glucan (BDG, 31.385) came back negative. Although BDG testing is often used in fungal diagnostics, its sensitivity can be limited in non-neutropenic patients and in cases of localized infection. Diagnostic and therapeutic ultrasound was carried out again. Multiple septations on the left side were observed with increased echogenicity. Five syringes of 10 c/c were taken for diagnosis and sent for cytology, which showed protein of 4.3 g, fluid albumin of 1.8 g/dl, fluid LDH of 712 IU/L, and fluid glucose of 79 mg/dL, which was evident for an exudative picture.

**Figure 3:**
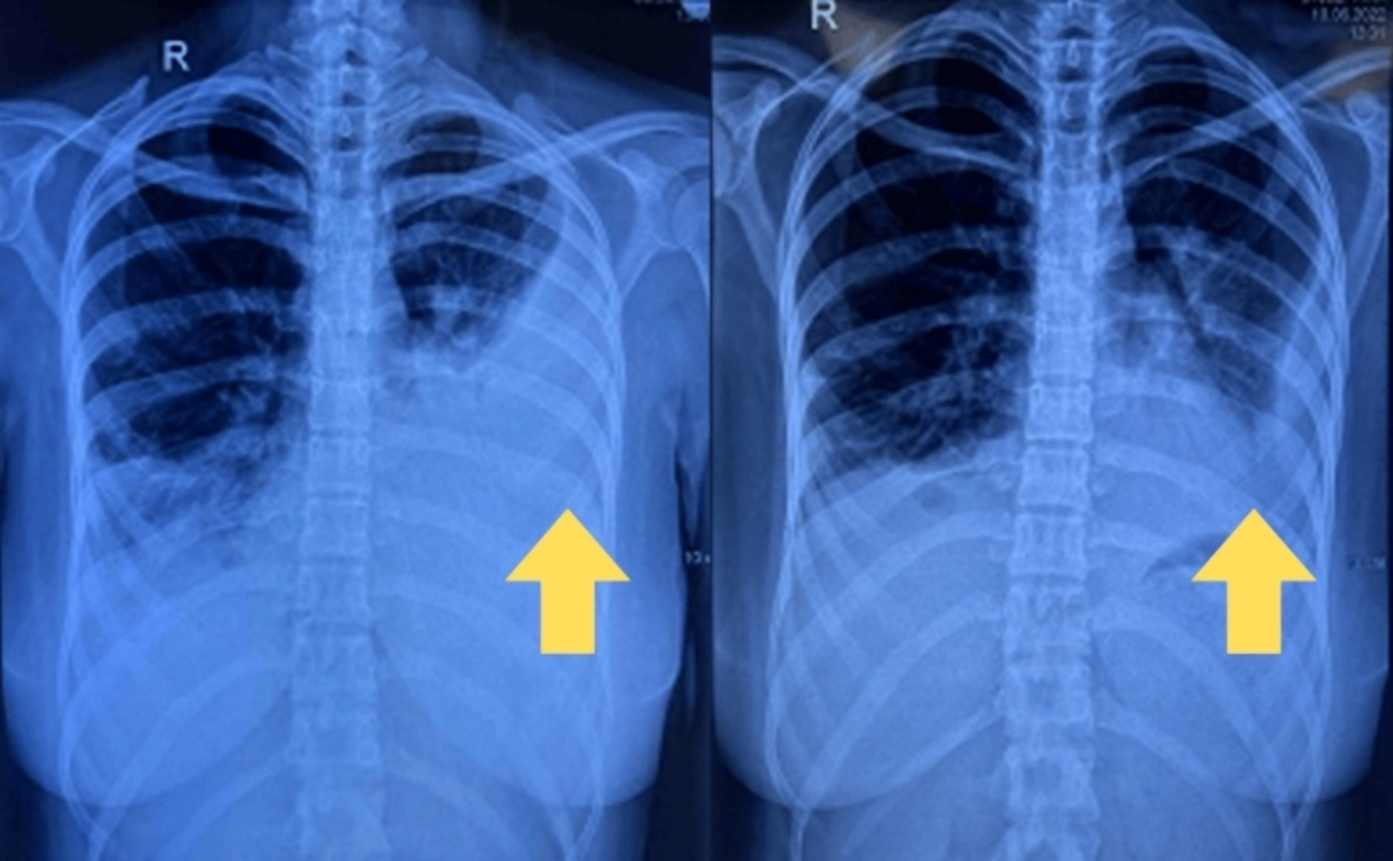
Serial chest radiography. (Left) Chest radiograph on day 13. (Right) Chest radiograph on day 23. Yellow arrow pointing to pleural effusion.

The patient was put on a voriconazole oral tablet. Meanwhile, she became afebrile, and her oxygen saturation progressively improved. There was a significant improvement in laboratory results and radiological investigations as well. She was finally discharged on the 28th day of admission, with advice to continue the antifungal medication until the next follow-up (Figure 3).

## Discussion

*Aspergillus* species cause a wide spectrum of illnesses in humans, including allergy, superficial infection related to local trauma, and invasive disease, of which acute IA affects severely immunocompromised persons [8,9]. IA is also becoming an important infectious disease in intensive care unit (ICU) patients without the classical risk factors (neutropenia, leukemia, hematopoietic stem cell transplantation), and the mortality is also devastating in these apparently less immunocompromised patients. *Aspergillus* spp. is isolated from lower respiratory patients, and in about half of these patients, this finding represents IA [10].

Moreover, positive cases of fungal co-infection with dengue illness have not been widely reported, although it is a mold found in hospitals and homes alike. One of the major contributors to that case would be the low number of cases of IPA diagnosed based on histopathological evidence of fungal growth.

We have come across three cases of IPA following dengue recovery. One of which was an immunocompetent patient at Kasturba Hospital, Manipal, Karnataka, India, and two immunocompromised patients at a center in Southern Taiwan [1,11].

The case reported in Karnataka indicated the patient had the same clinical findings as our patient, except she was severely hypoxemic and had to be mechanically ventilated and medically managed for ongoing sepsis. Our case had pericardial effusion as well as pleural effusion, wherein we isolated some exudative samples [12].

Our case, along with the cases in Taiwan, has already highlighted that even though the serum assay for galactomannan was negative, the butyrolactone (BTL) sample showed conidia and fungal hyphae, and tested positive for serum galactomannan [1]. These two cases in Taiwan were geriatric patients, immune-compromised, and had a poor prognosis. It is, however, observed in all the reported cases that the diagnosis of IPA was delayed, and initially thought to be pulmonary bacteremia. This led to precious time being lost and probably contributed to ICU admission and death in some cases.

Although there was no initial neutropenia, the late development of lymphopenia and thrombocytosis accurately sums up the conclusion that fungal co-infection, along with dengue, causes T cell depletion. That ensues and promotes IA along with incorrect medication administration, leading to apparent hypoxemia and sepsis.

All these cases very clearly point toward the fact that IA is now not just a diagnosis of immunocompromised but can occur with immunocompetent patients as well, and there should be a low threshold of IA suspicion, among patients being reported from areas of populate dense areas or those where aspergillosis mold is known to habituate or have been admitted in ICUs for dengue or other viral infections, particularly respiratory.

Additionally, histopathological examination with microbiological confirmation remains the gold standard in diagnosis (almost 97% specificity), but is only positive in 50%-58% of patients. In non-neutropenic patients, such as those recovering from viral infections like dengue, traditional serum biomarkers like galactomannan (GM) and (1→3)-β-D-glucan (BDG) exhibit reduced sensitivity. This limitation underscores the need for alternative diagnostic approaches. Recent studies have highlighted the utility of non-invasive biomarkers, including lateral flow assays (LFAs) and *Aspergillus* polymerase chain reaction (PCR), which offer improved sensitivity and specificity in detecting IPA, especially in ICU settings [11,13,14]. Although this has now been adapted with a bronchoalveolar lavage (BAL) and galactomannan staining to facilitate the diagnosis of IA, cases still face diagnostic delay. Our point is therefore to accentuate a better means of investigation, not dependent on invasive tests like biopsies and BAL. In this age of technology, with such apparent serological disturbances observed in the pattern of disease, a rapid, specific, and cost-effective modality should be developed.

## Conclusions

This case underscores the importance of maintaining a low threshold for suspecting invasive fungal infections in post-viral or critically ill patients, even those without traditional risk factors for immunosuppression. Clinicians should be alert to signs of clinical deterioration that are disproportionate to the primary diagnosis. While the diagnosis of dengue was supported by positive NS1 antigen serology and contextual epidemiological factors, the overlapping features with early sepsis and the absence of fluid leakage in imaging highlight the challenges in diagnosing DHF definitively in some cases. Diagnostic uncertainty should always be acknowledged, especially when managing complex cases in resource-limited settings.
